# Horizontal and vertical movements of Caribbean reef sharks (*Carcharhinus perezi*): conservation implications of limited migration in a marine sanctuary

**DOI:** 10.1098/rsos.160611

**Published:** 2017-02-15

**Authors:** Oliver N. Shipley, Lucy A. Howey, Emily R. Tolentino, Lance K. B. Jordan, Jonathan L. W. Ruppert, Edward J. Brooks

**Affiliations:** 1 Shark Research and Conservation Program, The Cape Eleuthera Institute, PO Box EL-26029, Rock Sound, Eleuthera, The Bahamas; 2 School of Marine and Atmospheric Sciences, Stony Brook University, Stony Brook, NY, USA; 3 Microwave Telemetry, Inc., 8835 Columbia 100 Parkway, Suites K & L, Columbia, MD 21045, USA; 4 Department of Renewable Resources, University of Alberta, Edmonton, Alberta, CanadaT6G 2H1

**Keywords:** connectivity, elasmobranch, behaviour, spatio-temporal movement, pop-up satellite archival tags

## Abstract

Despite the ecological and economic importance of the Caribbean reef shark (*Carcharhinus perezi*), little data exist regarding the movements and habitat use of this predator across its range. We deployed 11 pop-up satellite archival tags on Caribbean reef sharks captured in the northeast Exuma Sound, The Bahamas, to assess their horizontal and vertical movements throughout the water column. Sharks showed high site fidelity to The Bahamas suggesting Bahamian subpopulations remain protected within the Bahamian Shark Sanctuary. Depth data indicate that Caribbean reef sharks spent a significant proportion (72–91%) of their time above 50 m in narrow vertical depth bands, which varied considerably on an individual basis. This may be indicative of high site fidelity to specific bathymetric features. Animals exhibited three broadly categorized sporadic off-bank excursions (more than 50 m excursions) down to a depth of 436.1 m, which were more frequent during the night. These deeper excursions during night may be indicative of foraging in relation to prey on mesophotic reefs, as well as diel-vertically migrating prey from the deeper meso- and bathypelagic zones. These vertical movements suggest that Caribbean reef sharks can be significant vectors of ecosystem connectivity further warranting holistic multi-system management and conservation approaches.

## 1. Introduction

The Caribbean reef shark (*Carcharhinus perezi*) is a medium-bodied (maximum total length, 295 cm [1]) carcharhinid found across the tropical and subtropical western Atlantic, with a range extending from North Carolina to southern Brazil [2]. Individuals primarily associate with shallow neritic habitats, such as coral reefs and lagoons; however, they also perform extended vertical excursions into deeper water (greater than 200 m) around continental drop-offs and escarpments [3,4]. This species also exhibits high site fidelity and philopatry, with limited annual horizontal displacement [4–7]. Caribbean reef sharks are considered one of the major top predators on coral reefs throughout the wider Caribbean and are pivotal to the health, and subsequent resources provided by these ecosystems [4,7]. In addition to their ecological role as top predators, common sightings on recreational shark dives, especially within the great Caribbean, create a significant economic reliance upon healthy Caribbean reef shark populations [8,9].

Across the Caribbean, concern has grown over the health and vitality of shark populations as considerable declines have been observed in recent decades [10,11]. High human population density has been strongly associated with the increasing absence of sharks in highly fished systems such as coral reefs, and localized species extinctions have been observed in some locations [10]. Although Caribbean reef sharks show high relative abundance compared with other predators across their range [11], artisanal [12] and commercial fishing [13], as well as coastal development [12,14], threaten their populations. This is probably exacerbated by their tendency to move across relatively large spatial scales through a multitude of ecosystems including coastal reefs, open-ocean and deep-water habitats (greater than 200 m) [4]. These concerns have resulted in the listing designation of the Caribbean reef shark as ‘Near Threatened’ by the International Union for the Conservation of Nature (IUCN) [2], highlighting a critical need for greater study and conservation action for this species.

A common strategy employed by conservation managers to protect, maintain and regenerate the diversity and health of marine ecosystems worldwide is through the creation of marine protected areas (MPAs) [15–18]. Despite their potential benefits, the effectiveness of MPAs in protecting highly mobile fishes, such as sharks, remains poorly quantified, as many management areas are situated across sparsely populated areas away from human settlement [19,20]. The high mobility of sharks often precludes their effective management within a single jurisdictional boundary, and for species that migrate large distances to facilitate important aspects of their life history, efficient protection often requires significant transboundary cooperation across multiple exclusive economic zones (EEZs) [21–23]. However, such cooperation may be inhibited by marine protection measures that vary across jurisdictional boundaries [23,24]. In 2011, The Bahamas became the fourth nation to declare its entire EEZ (611 151.9 km^2^) a shark sanctuary outlawing commercial fishing and landing of any shark species [22,25]. Commercial longlining, which can have negative impacts on shark populations through incidental bycatch and subsequent stress-induced mortality [26,27], is therefore illegal in The Bahamas [22]. Such approaches have proved successful in protecting shark populations in some regions. For example, Robbins *et al*. [28] observed that shark abundance was an order of magnitude higher in no-entry management zones, compared with fished-reefs on the Great Barrier Reef, Australia. The Bahamas, therefore, offers a unique opportunity to quantify the movements and interactions of an apex predatory shark, which resides fully, or for part of its life history, within a single marine management zone [22,23].

Although the abundance, ecological and economic importance of Caribbean reef sharks in The Bahamas has been noted [18,27,29], their broader movement in relation to the Bahamian EEZ, and vertical habitat use in this region, remains poorly understood. Therefore, the degree to which these animals may travel outside of this MPA, and thus become vulnerable to exploitation elsewhere, is unknown. Here, we used pop-up satellite archival tags (PSATs), which have proved valuable in assessing shark movements [22,30–32], to examine horizontal and vertical movements exhibited by Caribbean reef sharks in The Bahamas.

## 2. Material and methods

### 2.1. Study area

The Exuma Sound is a Bahamian deep-water inlet of the Atlantic Ocean that separates the Exuma Cays (to the west) from Eleuthera and Cat Island (to the east). The sound is categorized by rapidly sloping margins at the bank-break, dropping from 30 to greater than 500 m, and reaching a maximum depth of 1600–2000 m in the central Sound [33]. The rapidly sloping bank-break is categorized by rugose limestone outcroppings, which provide a complex bathymetry [34] and transitions into a muddy silted benthos categorized by clastic turbidites [35].

Animals were captured at two locations (southwest Eleuthera and the Bridge) via stationary midwater longlines (see Brooks *et al*. [7] for detailed methodology) between January 2011 and June 2013, in the northeast Exuma Sound. Southwest Eleuthera is largely composed of continuous fringing coral reef and large coral heads, lying adjacent to a rapidly steepening continental drop-off. In addition, this location is proximal to shallower oolitic banks and sand flats north of Cape Eleuthera [7]. The Bridge is a shallow limestone bank (approx. 40 m deep) connecting southern Eleuthera to Cat Island, known to have a high abundance and diversity of large apex predators (E. Brooks 2010, unpublished data). Although the Bridge has similar biological and bathymetric properties to southwest Eleuthera, it does not offer such proximity to shallower banks and sand flats.

### 2.2. Pop-up satellite archival tag deployment

**Figure 1.**
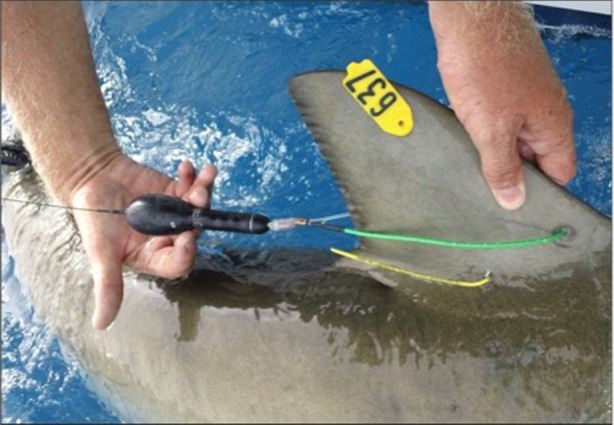
X-Tag attachment through the leading (anterior) edge of the first dorsal fin of a Caribbean reef shark. Conventional, yellow steel-headed dart tag is also visible.

Sharks were tagged with X-Tags (Microwave Telemetry, Inc., Columbia, MD, USA). A small pilot hole was made through the leading (anterior) edge of the dorsal fin using a sterile, stainless steel scalpel prior to tag attachment. Tags were then attached with a monofilament bridle (2 mm diameter) equipped with a heat-shrink chafe protector, which passed through the leading edge of the dorsal fin. Bridles were secured under the trailing edge using a copper crimp covered with surgical rubber insulation (figure 1). X-Tags recorded time-series depth (0.3–5.4 m resolution), temperature (0.16–0.23°C resolution) and light-level data. After the tag's predetermined deployment duration, it detached from the animal and floated to the surface. A subset of archived data was transmitted to the Argos-equipped satellite system. The release may be initiated prematurely if the constant pressure or maximum-depth emergency release mechanism is triggered. X-Tags can have high rate (HR) or standard rate (SR) programming. HR tags in this study were programmed for short deployments (30 days), providing a relatively high sampling rate (approx. 5 min intervals) but no location estimates. Unlike HR tags, SR tags provide daily light-based geolocation estimates with a best-possible error of ±1° for latitude and ±0.5° for longitude. Additionally, SR tags collect data at 2 min intervals, but only a subset of records is selected for transmission through the Argos system; the temporal resolution of the transmitted dataset (15–60 min) depends on the deployment duration. Transmitted SR depth and temperature data may contain delta limited records which do not capture the full range of vertical movement or temperature variations; however, all delta limited records were included in the analysis. In the event that a SR or HR X-Tag is recovered, the entire archived dataset (2 min records in SR tags and 5 min records in HR tags) may be extracted (http://www.microwavetelemetry.com/fish/).

### 2.3. Statistical analysis

#### 2.3.1. Horizontal movements

Daily geolocations obtained from SR X-Tags were processed with a state-space unscented Kalman filter with sea surface temperature (UKFSST) [36]. Daily sea surface temperatures (SSTs) recorded by the tag were estimated by daily maximum temperature values [37], and the NOAA Optimum Interpolation Sea Surface Temperature V2 dataset ([38]; http://www.esrl.noaa.gov/psd/) was used as the reference SST field. A post hoc bathymetric correction was applied to the filtered tracks [39] and implemented with the ‘analyzepsat’ package in R [37,40].

#### 2.3.2. General vertical habitat use

Visual assessment of time-series depth records suggested that individuals occupy consistent narrow regions of the water column, varying among individuals. Therefore, to further investigate individual depth occurrence, the density function in R was applied to the time-series depth records to identify high-use vertical depth bands, specifying a density threshold of 0.01 [41].

We employed generalized linear mixed-effect models (GLMM) to determine factors affecting the vertical habitat use of the Caribbean reef shark. Specifically, two models were constructed to predict the response variables of mean depth and count of depth records below 50 m. The 50 m isobaths was identified as a significant departure from shallow-water habitat as the edge of the bank in the Exuma Sound is typically marked by a near-vertical escarpment originating at 20–30 m depth. Consequently, a depth record of 50 m would indicate a 20–30 m departure from this well-defined bathymetric feature. Furthermore, the depth data generated by Chapman *et al*. [4] indicated that Caribbean reef sharks primarily resided at depths of less than 40 m. Prior to analysis, both the mean depth and deep dive counts were log-transformed because they were both right skewed (for mean depth, the log[*x *+ 1] transformation was applied to simplify interpretation of results). GLMM fixed effects included moon phase, diel period, season, sex, stretched total length (centimetres) and location of tagging (southwest Eleuthera or the Bridge). Moon phase data were acquired from the USA Naval Observatory (http://aa.usno.navy.mil/data/docs/MoonFraction.php), and the lunar illumination values were partitioned into five illumination factor levels (based on 20% increments). Daily sunrise and sunset times were estimated from UKFSST-filtered locations for SR tags and from the deployment locations for HR tags, allowing the assignment of diel period to each record. Dawn was defined as the 2 h period centred on sunrise, and dusk was defined as the 2 h period centred on sunset. Individual sharks were included as a random effect [42]. Because we investigated responses to cyclical patterns of moon phase, only individuals that had deployment durations greater than two times the length of a moon phase were included (i.e. 59 days; *n* = 5 individuals) (table 3).

GLMMs were implemented with the ‘lme4’ package in R [43]. We employed a backward elimination of non-significant effects to determine if a reduced model may better explain variation in depth, using the ‘lmertest’ package in R [44]. The fit of the full and reduced models was assessed by evaluating the corrected Akaike information criterion (AICc) and Bayesian information criterion (BIC) values. Model residuals were visually assessed to confirm the normal distribution and homoscedasticity of model residuals. Finally, we conducted family-wise Tukey comparisons to compare factor levels of significant fixed effects with the ‘multcomp’ package in R [45,46].

#### 2.3.3. Off-bank excursion analysis

To investigate behaviour off the Great Bahama Bank (greater than 50 m), individual off-bank excursions were extracted. Only the tags providing depth data at a temporal resolution of less than or equal to 5 min (*n* = 7) were considered for this analysis. An excursion (or dive event) was defined as a sequence of consecutive (greater than or equal to 1) depth records below the 50 m threshold. The beginning of the excursion was specified as the last record above 50 m immediately before the dive event, and the end of the excursion was the first record above 50 m immediately after the dive event [47]. A dive event was not accepted for the analysis if it contained temporal gaps (i.e. missing data). Given considerable variability observed in depth-versus-time profile shapes, dives were classified based on characteristic variables instead of shape. Specifically, five dive variables were selected: dive maximum depth (metre), duration of dive event (minutes), switch count (the number of times that the vertical direction changed over the course of a dive event), mean vertical descent rate (m s^−1^) and mean vertical ascent rate (m s^−1^). Within each excursion, descents and ascents were defined as all pairs of consecutive depth records exhibiting an increase and decrease in depth, respectively. Prior to clustering dive events based on similar characteristics, principal component analysis (PCA) was applied to the five log-transformed, scaled and centred dive variables in order to generate orthogonal components better suited for clustering procedures [48]. Components explaining the majority of variance and satisfying the latent root criterion were retained for k-means clustering [48,49]. To determine the optimal number of clusters, we considered *R*^2^ versus cluster number [50–52]. Spearman's rank correlation (*r*_s_) was used for reported correlations, and we specified a significance level of 0.05 for all statistical analyses.

## 3. Results

**Figure 2.**
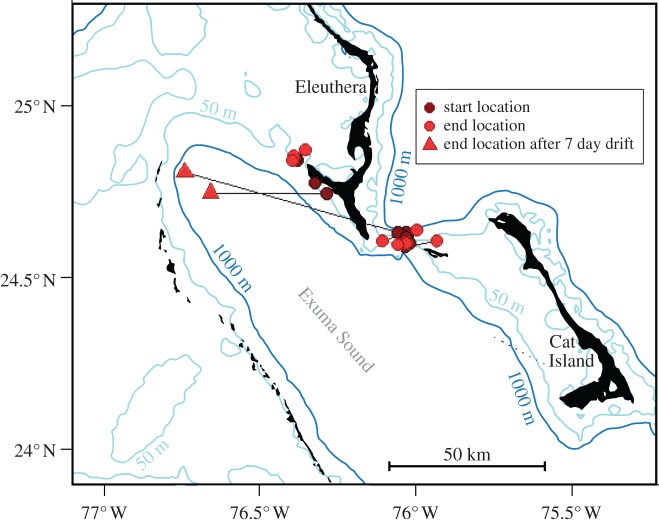
Map of tagging sites (start locations) and first Argos locations (end locations) connected by straight lines to illustrate the net displacement for each tracked Caribbean reef shark. Given the close proximity between start and end locations, the displacement lines are only evident in the two cases such that the tag reported after drifting for 7 days at the surface.

Eleven X-Tags (five HR, six SR) were deployed on mature Caribbean reef sharks (male = 5; female = 6) across the two sampling regions (table 1 and figure 2). Data were obtained from all tags, and five tags (three SR tags and two HR tags) were physically recovered, allowing for the extraction of their complete archived datasets (table 1). Data from tag 115972 implied post-release mortality by an unknown predator and was excluded from all further analyses. For this tag, a steady descent to approximately 1035 m was recorded immediately after release; the tag remained at this depth for approximately 18 h prior to vertical oscillations between approximately 400 m and depths of greater than 650 m beginning on a diel cycle. The time-paired depth and temperature records indicated a temperature delay with respect to the depth profile, supporting tag consumption [53]. The remaining 10 X-Tags provided 337 728 depth records (3068–173 861 records per individual), of which only 13 records were identified as delta limited values. Depth records ranged from the surface to 436.1 m, and all tags registered depth records greater than 100 m. Additionally, tags provided 336 625 temperature records (3068–173 861 records per individual). Temperature records ranged from 17.78 to 31.26°C, and only four records were identified as delta limited values.

**Table 1.** Summary information for Caribbean reef sharks tagged with HR and SR X-Tags; R indicates that tag was physically recovered.

ID	capture site	latitude (N)	longitude (E)	length (TL, cm)	sex	deployment date	X-Tag programing	deployment duration (days)	% data received
49969	SW Eleuthera	24.848	76.382	195	F	1 Sep 2011	HR	31	91
49970	SW Eleuthera	24.841	76.379	218	F	3 Sep 2011	HR	31	92
49971	the Bridge	24.588	76.032	182	M	11 Nov 2011	HR	30	35
49972	SW Eleuthera	24.848	76.382	196	F	1 Sep 2011	HR	31 (R)	100
49973	the Bridge	24.601	76.019	165	M	16 Nov 2011	HR	30 (R)	100
107800	the Bridge	24.588	76.032	183	F	10 Nov 2011	SR	243 (R)	100
115970	the Bridge	24.632	76.031	204	F	25 Nov 2012	SR	242	54
115971	the Bridge	24.630	76.058	180	M	16 Mar 2013	SR	145 (R)	100
115972^a^	SW Eleuthera	24.775	76.323	167	M	27 June 2013	SR	53 (R)	100
115973	the Bridge	24.616	76.028	182	F	25 Nov 2012	SR	242	62
115974	SW Eleuthera	24.746	76.284	175	M	28 June 2013	SR	176	73

^a^Presumed to be have been consumed.

### 3.1. Horizontal movements

The initial Argos-estimated position ranged between 1.1 and 71.6 km from the initial tagging site (figure 2). Tags released in the months of July, August, October and December. While most tags reported within 10 km of the tagging site, two tags (115971 and 115974) reported in the central Exuma Sound after drifting for seven days prior to reporting through the Argos system. Therefore, the net displacements of these two individuals are unconfirmed; however, it is likely these also popped-off adjacent to South Eleuthera which is directly down-current from the first Argos location. All other tags reported within one day of surfacing. The five SR tags provided 1065 light-based geolocations (145–243 daily locations per individual). The UKFSST-filtered tracks of four individuals indicated movement from Eleuthera to the northern side of Cuba below the Great Bahama Bank (maximum displacement of approx. 3° latitude) during the summer and autumn months. However, the UKFSST-estimated latitude standard deviation for these four tracks ranged between 1.48 and 4.13° N, and, therefore, filtered tracks confirmed that reef sharks did not embark on long-distance migrations extending beyond the error bounds of light-based geolocations.

### 3.2. General vertical habitat use

**Figure 3.**
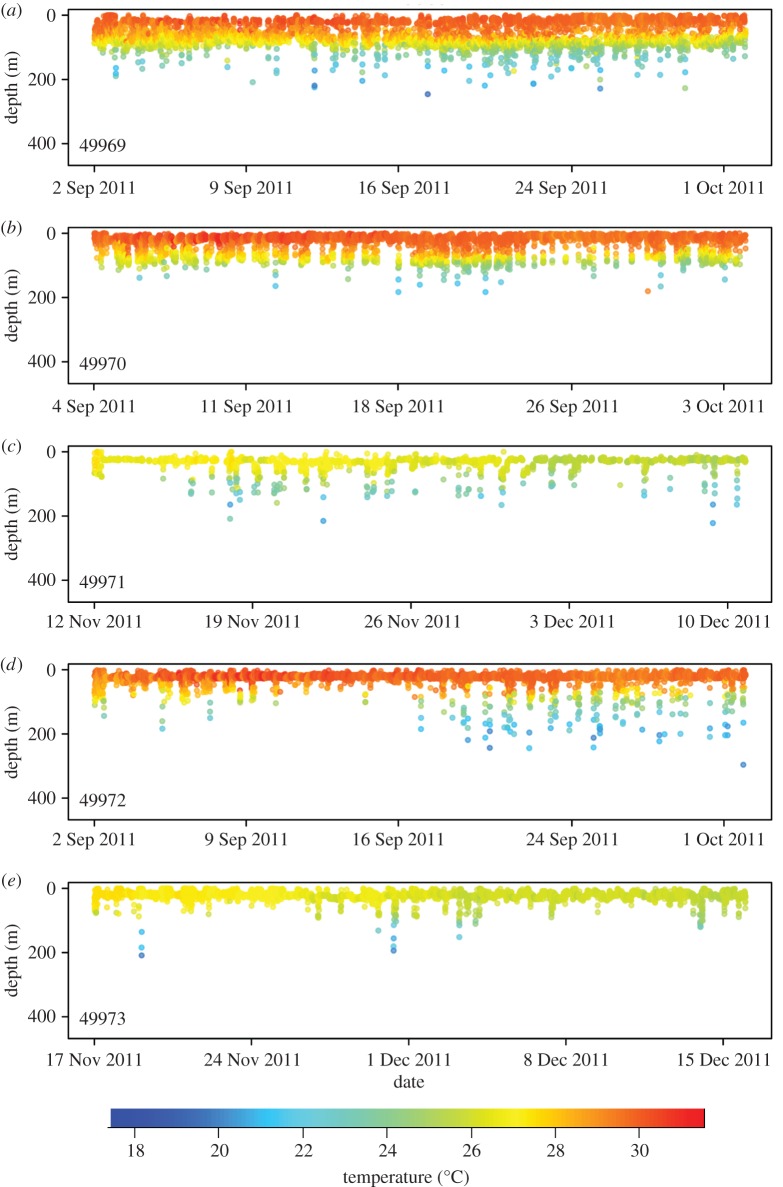
Depth-versus-time profiles coloured by concurrent tag-recorded temperature for each Caribbean reef shark tagged with an HR tag.

**Figure 4.**
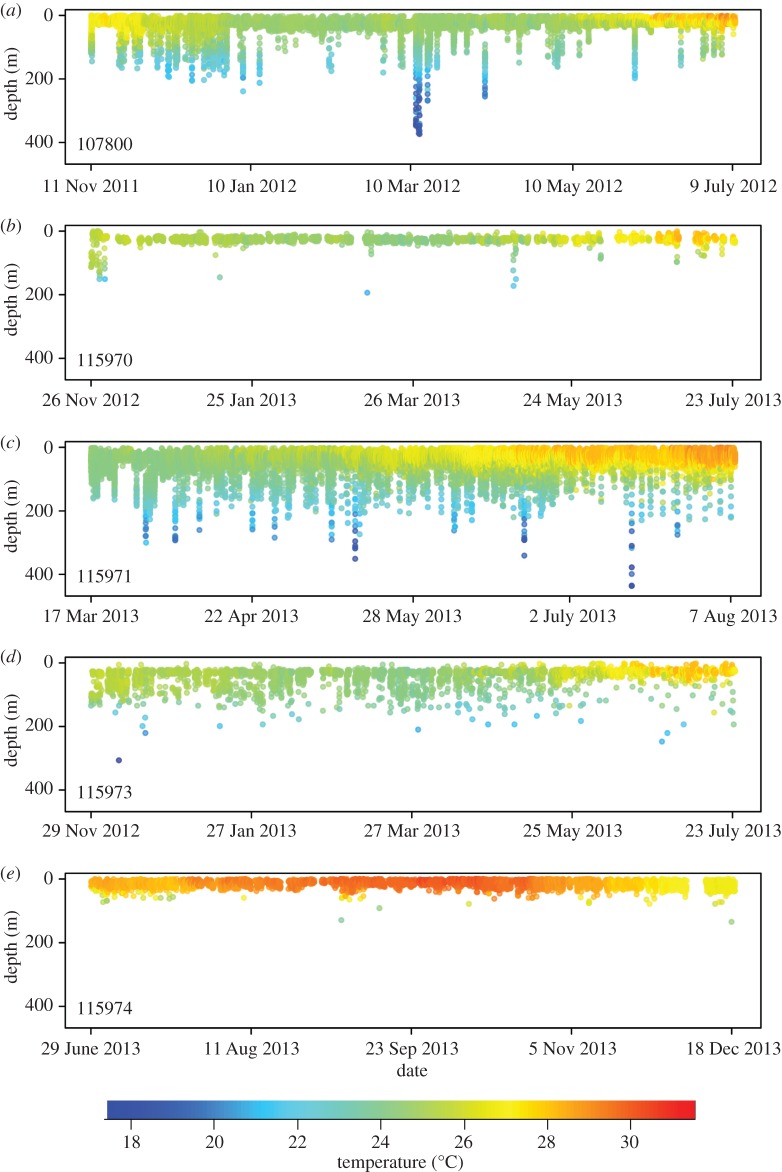
Depth-versus-time profiles coloured by concurrent tag-recorded temperature for each Caribbean reef shark tracked with an SR tag.

Individuals occupied narrow vertical bands, typically positioned in the top 50 m of the water column (table 2). The lower limit of high-use density depth bands from all individuals consistently corresponded to the bathymetric depth limitation of the steep drop-off on the edge of shelf bank. However, frequent excursions from the high-density depth bands were observed (figures 3 and 4), as all individuals recorded depths greater than 100 m. One individual (49969) exhibited a bimodal depth distribution, occupying a shallow vertical band and a deeper band below 50 m (figure 3 and table 2). The reduced mean depth model had lower AICc and BIC values compared with the full model, suggesting a more parsimonious and significant fit (table 3). Specifically, the mean depth within each period of the day was explained by the random effect of each individual and fixed effects of moon phase, diel period and season (table 3). However, post hoc analysis revealed that moon phase had no significant effect on depth occupied by individuals. For diel period, individuals occupied shallower mean depths during the day compared with any other period (*p* < 0.05). Additionally, individuals occupied shallower mean depths during the winter season compared with all other seasons (*p* < 0.001). Autumn and summer mean depth was greater than that observed during the spring season (*p* < 0.001). Finally, no difference between mean depths in the autumn and summer seasons was detected. The reduced GLMM predicting the number of deep depth records (i.e. greater than 50 m) had lower AICc and BIC values compared with the full model (table 3). The number of deep depth records within each diel period was explained by the random effect of each individual, and fixed effects of tagging location, sex, total length, diel period and season (table 3). Sharks recorded more deep records if individuals were either male or tagged at the Bridge (*p* < 0.001). Depth records greater than 50 m were more common at night than any other diel period and were more common during the day compared with dawn (*p* < 0.001). Finally, more deep records occurred during the winter than the summer (*p* < 0.05). However, a larger sample size is required to assess seasonal effects.

**Table 2.** Caribbean reef shark high-use vertical depth bands, defined as depth ranges having a density greater than a 0.01 threshold. (Time spent within the high-use areas is provided.)

ID	high-use depth band (m)	depth band range (m)	per cent time in depth band
49969	7.4–23.8, 65.6–92.1	16.4, 26.5	81
49970	0.9–25.3	24.4	73
49971	18.0–34.4	16.4	78
49972	11.2–28.7	17.5	85
49973	9.4–33.7	24.3	88
107800	10.2–34.4	24.2	98
115970	15.4–34.7	19.3	92
115971	15.0–38.8	23.8	85
115973	19.0–39.6	20.6	72
115974	0.44–27.5	27.1	91

**Table 3.** GLMM results. (Summary statistics for generalized linear mixed models predicting mean depth (depth) and number of deep dive events (deep) exhibited by Caribbean reef sharks. Shown for each full and reduced model are the degrees of freedom (d.f.), Bayesian information criterion (BIC) and corrected Akaike information criterion (AICc).)

model	d.f.	BIC	AICc
depth ∼ moon + season + period + sex + location + STL	16	1876.7	1858.9
depth ∼ moon + season + period	13	1862.4	1850.5
deep ∼ moon + season + period + sex + location + STL	16	7337	7308.1
deep ∼ season + period + sex + location + STL	12	7314	7290.2

### 3.3. Off-bank excursion analysis

Individuals with high-resolution datasets (*n* = 7) demonstrated 2118 excursions below 50 m, indicating the use of deeper habitats adjacent to the Great Bahama Bank (table 4). Depth-versus-time dive profiles exhibited highly variable shapes and durations. Nearly a quarter of off-bank excursions (522 dives, 24.6%) contained only one record below the 50 m threshold. Dive switch count ranged from 1 to 55 among dive events; however, the majority of excursions (77.2%) only contained one change in direction (switch count = 1), such that the time–depth profile appeared as a ‘V-shaped’ dive. Switch count correlated with the duration of the dive (*r*_s_ = 0.638, *p* < 2.2 × 10^−16^), indicating that longer-duration dives exhibited more vertical oscillations in the water column. The duration of the middle 50% of dive events ranged from 6 to 26 min, and the longest dive event lasted 6.7 h.

**Table 4.** Off-bank excursion statistics. (Statistics for depth excursions > 50 m for each Caribbean reef shark dataset with temporal resolution ≤ 5 min. Values in parentheses represent the interquartile range (IQR).)

ID	per cent records > 50 m	total dive count	median time between excursions (h)	maximum time between excursions (days)	transitory dive rate (dives h^−1^)	extended dive rate (dives h^−1^)	directed dive rate (dives h^−1^)
49969	59.6	300	0.6 (1.4)	0.5	0.07	0.25	0.08
49970	18.0	143	2.2 (5.0)	1.1	0.04	0.12	0.02
49971	10.5	54	2.1 (14.4)	6.1	0.04	0.01	0.02
49972	5.5	137	1.6 (5.3)	1.7	0.10	0.03	0.06
49973	1.9	67	2.7 (14.9)	2.3	0.07	0.01	0.01
107800	1.1	227	3.6 (22.3)	17.3	0.02	0.00	0.02
115971	5.6	1190	0.9 (3.0)	2.6	0.22	0.02	0.09

PCA yielded two leading principal components explaining 46.6% and 36.8% variation. Cluster analysis applied to these two components provided three clusters, explaining 60.9% of the variation among dives. The first cluster, termed the ‘transitory dive cluster’, represented relatively shallow and short-duration dive events with low switch count values and moderate vertical velocities (table 5). The second cluster represented moderate-depth dive events with long durations, exhibiting the largest range in switch count and the slowest vertical velocities (table 5). Consequently, this group was termed the ‘extended dive cluster’. The third cluster, termed the ‘directed dive cluster’, represented deep (and relatively short-duration excursions) with moderate switch count values and the fastest vertical velocities (table 5). Dive frequency within the three dive types was highly variable among individuals (table 4). Tag 115971 (male) recorded the highest rates of transitory dives (table 4). Extended dives were dominated by two female individuals (49969 and 49971) (table 4).

**Table 5.** Off-bank excursion cluster statistics. (Summary of Caribbean reef shark dive characteristics for each off-bank (> 50 m) excursion cluster, including the median (interquartile range) and range.)

cluster	dive maximum depth (m)	dive duration (min)	dive switch count	dive mean descent rate (m s^−1^)	dive mean ascent rate (m s^−1^)
1. transitory	60.2 (16.5)	6.1 (6)	1 (0)	0.112 (0.104)	0.103 (0.080)
excursions	50.1–106.6	4.0–33.4	1–3	0.002–0.477	0.011–0.464
2. extended	90.1 (22.2)	47.6 (42.8)	3 (4)	0.043 (0.034)	0.045 (0.030)
excursions	50.1–370.8	4.0–400.3	1–55	0.005–0.202	0.003–0.148
3. directed	131.5 (57.8)	19.1 (14.6)	1 (2)	0.222 (0.206)	0.166 (0.096)
excursions	76.7–436.1	4.0–104.3	1–13	0.042–1.187	0.046–0.627

## 4. Discussion

The Exuma Sound and its neighbouring mosaic of coastal ecosystems allowed examination of the horizontal and vertical behaviour of a mobile predator, occurring at the interface between the narrow near-shore shelf and open-ocean/deep-water habitats. Sharks tagged off Eleuthera did not embark on long-distance migrations and remained mostly resident within the protective boundaries of the Bahamian EEZ. The close proximity between deployment and pop-off locations after varying deployment durations, and at different times of year, suggests that individuals did not travel far from their initial tagging locations, potentially remaining resident to the waters off South Eleuthera. This is further supported by observations at other locales, such as Chapman *et al*. [4], where a single individual was tracked approximately 30 km over 150 days. Yet, the possibility of small-scale migrations should not be discounted, and although the direction of movement away from Eleuthera remains inconclusive, the general timing of this behaviour (indicated by UKFSST-filtered tracks) may be relevant, as it coincides with reproductive activity of Caribbean reef sharks at other Bahamian locales [8]. Although light-based geolocation estimates proved somewhat limited in their ability to discern finer-scale movements in relation to the EEZ boundary, this could be achieved in future studies through the use of passive telemetry [54], or chemical tracer approaches such as stable isotope analysis [55].

Healthy, less-impacted reefs tend to have larger proportions of biomass occupying higher trophic guilds; therefore, the presence of sharks is a good indicator of overall reef health [56,57]. In The Bahamas, a protected population of Caribbean reef sharks can contribute to both ecological and economic sustainability by encouraging ecosystem diversity and sustaining ecotourism vital to the Bahamian economy (i.e. shark diving). However, the relatively localized movements, if uniform across subpopulations, may have greater implications for individuals in unprotected regions. For example, the bottom longline fisheries of Columbia target Caribbean reef sharks for their skin, oil and fins [2] and may be contributing to significant population declines within unprotected subpopulations. Although this was beyond the scope of this study, assessing Caribbean reef shark movements across multiple locations will help identify and facilitate more appropriate management strategies within and between nations.

Caribbean reef sharks occupied narrow depth bands, typically in the top 50 m of the water column, with the bottom edge of the bands corresponding to the depth of edge of the bank. Vertical habitat use appeared variable among individuals, suggesting that depth-use may be governed by high site fidelity to a specific location, habitat or bathymetric feature. Brooks *et al*. [7] previously observed minimal horizontal displacement (approx. 1.7 km) in recaptured individuals from the Exuma Sound, suggesting that Caribbean reef sharks may remain extremely resident to a small area of reef. One individual (49969) demonstrated a bimodal distribution occupying two vertical bands, one in the top 50 m and another between 66 and 92 m. Because this pattern was only observed in one individual, we do not believe the bimodal distribution represents depth-mediated sexual behaviour, and more likely indicates habitat use across two distinct bathymetric features along the drop-off. Individuals resided shallowest during the day compared to any other diel period and could reflect movement of individuals between the shallow mosaic of coastal habitats (oolitic banks, coral reefs, seagrass beds, etc.) surrounding the sampling location, which may offer suitable protection from predators throughout the day [4]. Seasonally, individuals inhabited shallowest mean depths during the winter, which could reflect behavioural thermoregulation, as assumed in other ectothermic elasmobranchs [22,32,58].

Caribbean reef sharks exhibited frequent, yet sporadic, off-bank excursions (every few hours) beyond the high-use vertical bands, and particularly, beyond 50 m. These vertical excursions occurred more frequently during the night, when sharks may forage more actively [59]. The diet of Caribbean reef sharks is reportedly broad (teleosts and cephalopods [60]); therefore, individuals could be exploiting a diverse prey pool on mesophotic reefs (approx. 50–130 m) extending down the drop-off, as seen in the closely related Galapagos shark (*Carcharhinus galapagensis*, [61]). Sharks may then make even deeper excursions (greater than 130 m) to exploit meso- and bathypelagic prey, which move to more accessible depths during the night [62]. The increased frequency of vertical excursions during the winter, compared to summer, could relate to food pulses associated with deepening of the mixed layer. Alternative hypotheses explaining the functionality of large vertical movements have been postulated for highly mobile sharks [47], such as navigation [63], energy conservation [64] and interactive behaviour [65]. Although such explanations cannot be discounted in this study, evidence for foraging-based movements appears most plausible, certainly for coastal shark species, and therefore alternative solutions are discussed no further. The maximum time between off-bank excursions (greater than 50 m) varied among conspecifics, where one individual (49969, female) did not spend any longer than 12 consecutive hours in shallow waters (less than 50 m), yet another (107800, female) spent over two consecutive weeks (17 days) in shallow waters (less than 50 m). Again, this suggests an association of each individual with a unique locale, and preference for a specific bathymetric feature or section of reef.

Off-bank excursions were further classified into three clusters: transitory, extended and directed excursions; however, the functionality of these profiles was difficult to determine given variability in individual behaviour and low sample size. Transitory excursions represent short forays below the 50 m isobar that possibly indicate frequent off-bank excursions without directly exploiting deeper waters. The characteristics of directed excursions (i.e. high vertical velocity and deep depths) suggest that these behaviours are deliberate movements into the deeper waters, potentially representing active prey-following behaviour [66], which would be required to exploit deeper prey pools. Extended excursions, primarily demonstrated by two females tagged at southwest Eleuthera, showed that reef sharks can make prolonged use of deeper waters near 100 m. This may reflect use of specific mesophotic coral reef habitats, which represent important prey resource pools for coastal requiem sharks in tropical and subtropical locales [61]. The strong association of Caribbean reef sharks with neritic environments and its typically opportunistic, broad diet [60] suggest that individuals exhibit foraging plasticity, whereby energetic requirements are fulfilled by feeding across multiple ecosystems (i.e. coral reefs and open ocean/deep-water). McCauley *et al*. [67] observed this behaviour in grey reef (*Carcharhinus amblyrhynchos*) and blacktip reef sharks (*Carcharhinus melanopterus*) at Palymyra Atoll, Pacific Ocean, where individuals relied on both coastal and pelagic prey items. It is likely Caribbean reef sharks may behave similarly. Such observations, therefore, have vital implications for holistic management approaches, as Caribbean reef sharks probably facilitate vital connectivity between coastal, mesopelagic and bathyal habitats.

In conclusion, the limited movements of Caribbean reef sharks suggest reduced interactions of this subpopulation with extrinsic fishing pressure, possibly sustaining ecological and economic vitality in The Bahamas. The highly variable off-bank excursions observed among individuals highlight the inherent complexities associated with vertical habitat use of coastal shark species, whereby deep-water ecosystems may serve as an important prey pool with Caribbean reef sharks serving as an important vector facilitating connectivity between shallow neritic and mesophotic (50–130 m) coral reefs, and deep-water (greater than 200 m) ecosystems.

## Data accessibility

Data published in this manuscript are available online through Dryad (http://dx.doi.org/10.5061/dryad.cm184) [69].
